# Carcinoma of the posterior wall of the hypopharynx: surgical treatment with larynx preservation

**DOI:** 10.1016/j.bjorl.2020.05.013

**Published:** 2020-06-15

**Authors:** Bora Başaran, Selin Ünsaler

**Affiliations:** a Istanbul University, Faculty of Medicine, Department of Otolaryngology, Istanbul, Turkey; b Koç University, School of Medicine, Department of Otolaryngology, Istanbul, Turkey

**Keywords:** Carcinoma, Posterior wall of the hypopharynx, Pharyngeal reconstruction, Laryngeal preservation

## Abstract

**Introduction:**

Posterior pharyngeal wall is the most rare subsite for hypopharyngeal carcinomas. Because of its rarity, there are few studies published in the literature specifically concerning posterior pharyngeal wall carcinoma.

**Objectives:**

To report our functional results in patients with the carcinoma of the posterior wall of the hypopharynx after surgical treatment by resection via a lateral or infrahyoid pharyngotomy approach, with the preservation of the larynx and reconstruction with a radial forearm free flap.

**Methods:**

The study included 10 patients who underwent surgery for a carcinoma of the posterior wall of the hypopharynx over a 6 year period. The associated postoperative morbidity was investigated and functional results were analyzed.

**Results:**

Nine patients had T3 lesions and one patient had a T2 lesion. The preferred approach to access the hypopharynx was a lateral pharyngotomy in 5 patients and lateral pharyngotomy combined with infrahyoid pharyngotomy in 5 patients with superior extension to oropharynx. The pharyngeal defects were reconstructed successfully with radial forearm free flaps. Four patients received adjuvant radiotherapy only, and 4 patients with N2b and N2c neck diseases received adjuvant chemoradiotherapy. The mean duration of hospitalization was 15.6 days (range, 10–21 days). All patients achieved oral intake in a median time of 74 days (range, 15–180). Decannulation was achieved in all patients and the median time for decannulation was 90 (range, 21–300 days). The mean followup duration was 38.3 months (range, 10–71 months) and 8 patients survived. One patient died due to regional recurrence in the retropharyngeal lymph nodes and 1 patient died due to systemic metastasis.

**Conclusion:**

Primary surgery is still a very effective treatment modality for the carcinoma of the posterior wall of the hypopharynx and does not permanently compromise the swallowing and laryngeal functions if pharyngeal reconstruction is performed with a free flap.

## Introduction

Squamous cell carcinoma of the hypopharynx represents only 3%–5% of all squamous cell carcinomas of the head and neck region^1^, ^2^ and posterior pharyngeal wall, is the most rare subsite for hypopharyngeal carcinomas.^3^ Because of its rarity, there are few studies published in the literature specifically concerning Carcinoma of the Posterior Wall of the Hypopharynx (CPWH). Primary surgery and postoperative Radiotherapy (RT) remains an important tool in the treatment of CPWH, but postoperative morbidity and reconstructive challenges of the surgical defect, which impacts swallowing, speech and respiration, has an important influence on choosing the therapeutic approach. In this study, we aimed to report our surgical experience in the treatment of advanced tumors by resection via a lateral or an infrahyoid pharyngotomy approach with laryngeal preservation and reconstruction of the pharyngeal defect with radial forearm free flaps. We also reviewed the literature for current trends in the treatment of these tumors.

## Methods

The medical charts and pathology reports of patients who underwent partial pharyngectomy with laryngeal preservation for posterior wall of the hypopharynx squamous cell carcinoma, in a tertiary university hospital between January 2013 and October 2018, were retrospectively reviewed. All of the patients were evaluated by the tumor board, consisting of radiation oncologists, medical oncologists, head and neck surgeons and a radiologist. The patients were informed about Chemoradiotherapy (CRT) protocols as an alternative treatment modality and the decision for surgical treatment was made regarding the patient preferences. The patients were staged according to the American Joint Committee on Cancer (AJCC) 2010 staging guidelines.^4^ Preoperative imaging with contrast MRI was performed to evaluate the potential tumor invasion of adjacent structures such as larynx and prevertebral fascia. The sagittal sections were helpful in assessing the vertical extension of the tumor, which is critical in choosing the surgical approach. A PET-CT scan was routinely performed to assess regional and distant metastasis. Patients in good health, without involvement of the prevertebral fascia, laryngeal structures, apex of the pyriform sinus or postcricoid area, and without distant metastasis were selected for the surgery. The patients included in the study had received no prior RT or surgical treatment for other head and neck cancer, and all of them were operated by the same surgical team.

The parameters which were retrospectively reviewed were nasogastric tube/gastrostomy tube removal time, decannulation time, donor and recipient site morbidities, hospitalization duration and postoperative complications. All patients except 2 who died before the study, were also asked to complete the Eating Assessment Tool (EAT-10) (Table 1) and the Functional Oral Intake Scale (FOIS) (Table 2) for the evaluation of the swallowing^5^ at the time of last followup visit. In EAT-10, the patients are asked to answer 10 questions by scoring each question between 0 and 4 (0: No problem, 4: Severe problem) and the total score is the sum of all. In FOIS, the patients are asked to score their quality of swallowing by choosing the most appropriate statement in the scale, numbered between 1 and 7.

**Table 1:** Functional Oral Intake Scale (FOIS).

Functional oral intake scale	Levels
No oral intake.	1
Tube dependent with minimal/inconsistent oral intake.	2
Tube supplements with consistent oral intake.	3
Total oral intake of a single consistency.	4
Total oral intake of multiple consistencies requiring special preparation.	5
Total oral intake with no special preparation, but must avoid specific foods or liquid items.	6
Total oral intake with no restrictions.	7

Levels 1‒3: tube dependent; Levels 4‒7: total oral intake.

**Table 2:** Eating assessment tool ‒10.

Circle the appropriate response	0 = No problem			
	4 = Severe problem			
1. My swallowing problem has caused me to lose weight.	1	2	3	4
2. My swallowing problem interferes with my ability to go out for meals.	1	2	3	4
3. Swallowing liquids takes extra effort.	1	2	3	4
4. Swallowing solids takes extra effort.	1	2	3	4
5. Swallowing pills takes extra effort.	1	2	3	4
6. Swallowing is painful.	1	2	3	4
7. The pleasure of eating is affected by my swallowing.	1	2	3	4
8. When I swallow food sticks in my throat.	1	2	3	4
9. I cough when I eat.	1	2	3	4
10. Swallowing is stressful.	1	2	3	4
Total EAT-10 =				

### Surgical technique

**Figure 1:**
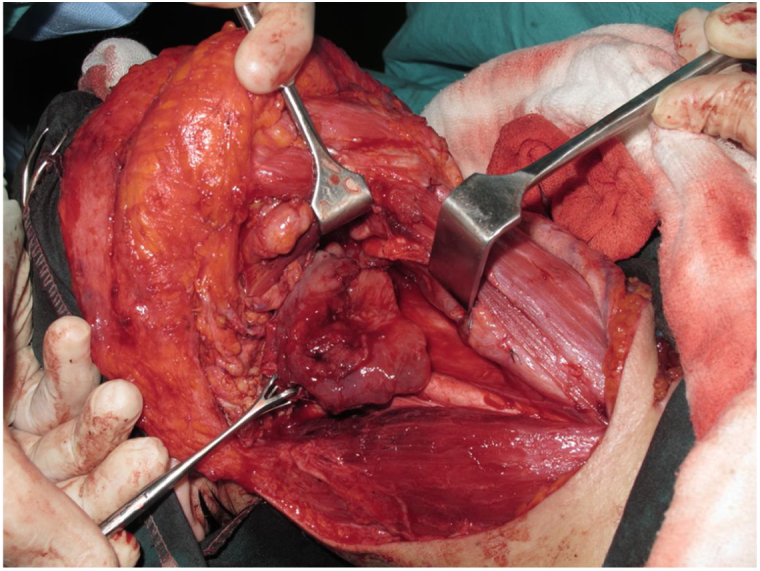
Tumor resected via lateral pharyngotomy.

**Figure 2:**
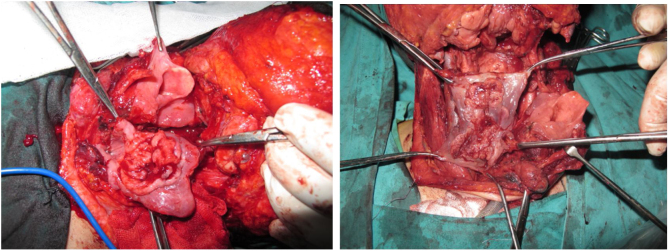
(a‒b) Tumors resected via combined lateral and infrahyoid pharyngotomy.

**Figure 3:**
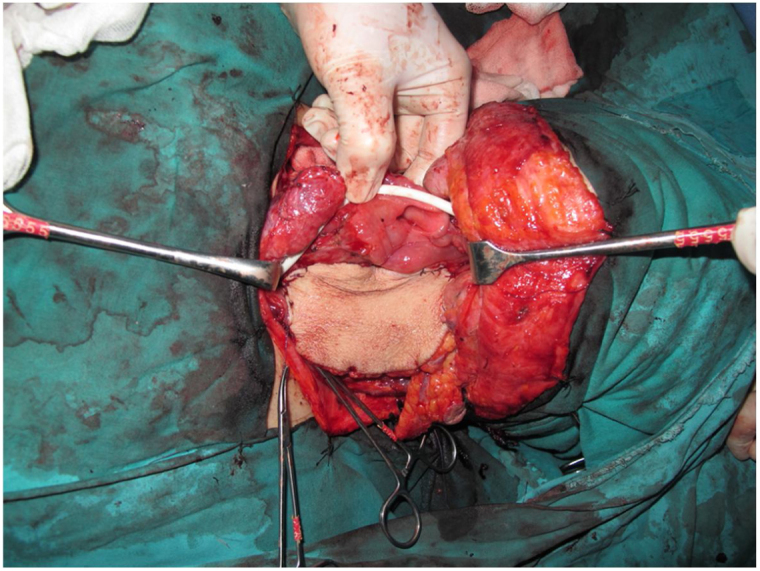
Reconstruction of the pharyngeal defect with radial forearm free flap.

Tracheotomy is routinely performed at the beginning of the operation. The prevertebral fascia is explored and assessed for involvement by the tumor, and then bilateral neck dissections are completed before starting pharyngectomy. The inferior constrictor muscle is divided from the thyroid cartilage on the side where the tumor is far from the Pyriform Sinus (PS) and the PS is released bluntly from the thyroid cartilage. After the tumor is palpated from outside of the PS, pharyngotomy is performed through the lateral wall of the PS and the incision is extended towards the esophagus to improve the exposure. The larynx is then retracted anteriorly (Fig. 1). Once the surgical borders are determined, superior and inferior pharyngeal incisions are created and the tumor is excised in an en-bloc fashion by a release from the contralateral PS as the final step. In case of oropharyngeal extension of the tumor, lateral pharyngotomy may not be sufficient for the exposure of the superior margin; it can be combined with an infrahyoid pharyngotomy (Fig. 2 a–b), which provides a wider exposure and easier flap inset, but also has the disadvantage of sacrifice of the superior laryngeal pedicle. In case the infrahyoid pharyngotomy is performed, the superior laryngeal nerve on the contralateral side must be meticulously preserved. After the tumor ablation, frozen sections from the mucosal borders are taken and once the surgical borders are confirmed to be clear of tumor, a radial forearm free flap is harvested with dimensions in accordance with the resection site requirements (Fig. 3). Additionally, an external monitor flap, which is vascularized by the same pedicle, is harvested to be sutured to the neck skin.

## Results

Of the 11 patients, one patient was excluded from the study due to short followup time. Of the remaining 10 patients, 6 were female and 4 were male; ages ranging from 37 to 68 years (mean 54.6‒9.85 yr). The mean follow up duration was 40.5 months (range, 14–75 months; SD ± 22.72 months).

In 5 of the patients, the superior border of the tumor extended above the level of the hyoid involving the oropharyngeal posterior wall, and in 1 patient the unilateral pyriform sinus was also involved. Nine patients had T3 tumor regarding the dimensions of the tumor and one patient had a T2 tumor. The preferred way to access the hypopharynx was lateral pharyngotomy in 5 patients and lateral pharyngotomy combined with infrahyoid pharyngotomy in 5 patients who had oropharyngeal extension. In all except 2 patients, bilateral posterolateral selective neck dissections were performed including levels II‒V; in 2 patients a unilateral modified type 1 radical neck dissection was performed on one side because of the radiological and clinical signs of extranodal extension of lymph node metastasis.

The surgical borders were free of tumor in all of the specimens. The neck diseases were 4pN0, 1pN1, 1N2a, 2pN2b, 2pN2c and except for 2 patients with T3N0 and T3N1 disease who rejected RT, all received adjuvant therapy. 3 patients with pT3N0 and 1 patient with pT3N2a disease received adjuvant RT alone and the other 4 patients with advanced neck disease received adjuvant CRT.

Postoperatively there was no complications with high morbidity. Two patients were returned to the operating room because of hematoma in the neck on the second postoperative day, which was controlled without a flap pedicle compromise. In 2 patients, partial necrosis of the split thickness skin graft at the donor site occurred. All of the radial forearm free flaps survived successfully and no salivary fistula or pharyngeal stenosis was observed in any of the patients.

### Oncological results

Of the 10 patients, 2 died due to disease. One of these 2 patients had presented with T3N0 disease and also received adjuvant RT, but developed regional recurrence in retropharyngeal lymph nodes bilaterally and died within 18 months of the operation. The other patient had T3N2c disease and received adjuvant CRT, but died due to extensive pulmonary and other systemic metastases within 25 months of the operation. No local recurrence occurred in any of the patients. The detailed clinical data of the patients are given in the Table 3.

**Table 3:** Summary of the each patient’s disease, treatment and survival status.

Patıent	Age	Sex	Tumor locatıon	Approach	TNM	Adjuvant therapy	Tube removal	Decannulatıon	Hospitalızatıon	Survival	Follow-up (months)	EAT-10 and/ FOIS scores
1	56	F	HPW	LP	T3N0	RT	90 (TPEG)	300	14	Alive	75	7/ 7
2	62	M	OPW + HPW	LP + IHP	T3N0	- (patient refused)	60	21	14	Alive	72	9/5
3	68	F	OPW + HPW	LP + IHP	T3N0	RT	90 (TPEG)	90	20	Died due to retropharyngeal lymph node metastasis	18	‒
4	38	F	OPW + HPW	LP + IHP	T3N1	-(patient refused)	60	45	10	Alive	63	7/ 7
5	59	F	Pyriform sinüs + HPW	LP	T3N2B	CRT	15	60	10	Alive	51	7/ 7
6	53	F	HPW	LP	T3N2B	CRT	150 (TPEG)	120	21	Alive	41	10/ 5
7	59	M	HPW	LP	T3N2C	CRT	42	90	19	Died due to pulmonary and systemic metastasis	25	‒
8	37	F	HPW	LP	T3N0	RT	30	90	20	Alive	28	9/ 6
9	57	M	OPW + HPW	LP + IHP	T3N2A	RT	120 (TPEG)	90	14	Alive	18	10/6
10	57	M	OPW + HPW	LP + IHP	T3N2C	CRT	180 (TPEG)	90	14	Alive	14	11/6

HPW, Hypopharynx Posterior Wall; OPW, Oropharynx Posterior Wall; LP, Lateral pharyngotomy; IHP, Infrahyoid Pharyngotomy; TPEG, Temporary Percutaneous Endoscopic Gastrostomy; EAT-10, Eating Assessment Tool; FOIS, Functional Oral Intake Scale.

### Functional results

Tracheostomy was performed routinely at the beginning of the operation. All the patients were decannulated within a median decannulation time of 90 days (range, 21–300 days). The shortest decannulation times (21 days and 45 days) were in two patients who did not receive RT. All the other patients were decannulated in accordance with the resolution of local edema after having completed RT. Oral intake time was determined as the gastrostomy tube removal or NGS removal time. Five patients who could not achieve oral intake within 2 months underwent percutaneous endoscopic gastrostomy, which were all closed later. All of the patients achieved oral intake within a median time of 75 days (range, 15–180 days). The mean duration of hospitalization was 15.6 days (range, 10–21 days, SD = 4.11). The mean EAT-10 score was 8.75 (range, 7–11, SD = 1.58). In the results of FOIS, two patients scored their status of swallowing 5; three patients scored 6; three patients scored 7 (Table 1).

## Discussion

Hypopharyngeal carcinoma has a very poor prognosis compared to other head and neck tumors due to an advanced stage of disease at the time of diagnosis and high rates of regional and distant metastasis during followup.^6^ The posterior pharyngeal wall is the least frequent subsite for hypopharyngeal carcinoma with the pyriform sinus being the most frequent and the postcricoid region the second.^3^ Because of the rarity of the disease, there is no standard treatment established by prospective randomized studies which could compare the superiority of different therapeutic modalities. The majority of the patients receive CRT either as a primary treatment modality or adjuvant therapy. The posterior pharyngeal wall is not an established subsite for p16 positive squamous cell carcinoma, thus surgery still has an important role as primary treatment.^6^ In the literature, the incidence of p16 positivity in the posterior wall of the oropharynx is between 0% and 19% in different studies.^7^, ^8^ P16 positive tumors originate from the reticular epithelium of the lymphoid tissue of Waldeyer’s ring, which is the reason for its high incidence in the tonsillar region and base of tongue. Additionally, unlike oropharyngeal cancer there is no association between p16 positivity and better survival outcomes in hypopharyngeal cancer.^9^, ^10^ Consequently, the treatment strategy is determined by the the combination of the experience of the clinicians and patients’ preferences. Although the prognosis is poor, the goal of the treatment should be providing the highest quality of life while achieving disease control. Therefore, laryngeal preservation with an intact functionality is an important issue while planning the treatment.

In the last two decades, there has been an increasing trend towards CRT for the treatment of larynx and hypopharynx cancers,^6^, ^11^ and in some retrospective studies retrospective studies, it is shown that this did not lead to worse survival outcomes for hypopharyngeal cancer, unlike that of laryngeal cancer, which has significantly declined.^11^, ^12^ CRT protocols had been reported to have similar outcomes with surgery and postoperative radiotherapy for hypopharyngeal cancer.^13^, ^14^ However, Kuo et al. published a retrospective study in 2014, which represents the current trend in hypopharyngeal carcinoma treatment in US. In this study, multivariate analysis adjusted for patient and tumor characteristics demonstrated that survival outcomes still differ with treatment modalities and the best 5 year overall survival rate was obtained with combined surgery and radiotherapy as 34.5%. The authors concluded that these findings suggest a survival advantage of combined treatment with surgery and adjuvant radiation.^11^ Moreover, in a study specifically about posterior pharyngeal wall carcinomas,^15^ De Felice et al. reported that 5 year local control was significantly higher in the primary surgery group compared with RT/CRT in both T1‒T2 (96.7% vs. 55.8%) and T3‒T4 diseases (73% vs. 52.7%). A significant Overall Survival (OS) benefit for 5 year was obtained with surgical treatment compared to RT/CRT in T1‒T2 diseases (63.8% vs. 39.7%), whereas OS in T3‒T4 diseases were not significantly different between surgery and RT/CRT arms (29.5% vs. 20.3%).

CPWHs without involvement of the laryngeal structures at the time of diagnosis are amenable to surgical treatment with an acceptable postoperative morbidity, since the larynx can be preserved with good functionality. There are 3 major concerns which should be considered by the surgeon: approach to the tumor with laryngeal preservation, resection of the tumor with safe margins, and finally, maintaining laryngeal and hypopharyngeal functions, which often requires reconstruction. The surgical approaches include an open approach with lateral pharyngotomy alone or combined with infrahyoid horizontal pharyngotomy as preferred in our series or suprahyoid pharyngotomy^16^; and transoral procedures, either with Transoral Laser Microsurgery (TLM)^17^ or with Transoral Robotic Surgery (TORS).^18^ When the open approach is preferred, the pharyngotomy site is determined depending on the extent of the tumor. Infrahyoid pharyngotomy alone does not provide exposition down to the level of the cricoid, so lateral pharyngotomy was our preferred approach. If lateral pharyngotomy is not sufficient to reach the superior margins of the tumor, the incision can be extended superiorly to the level of the hyoid. Another separate incision at the suprahyoid level may be preferred, but it was not performed in any of the patients in this study.

The transoral approaches may have the advantage of not necessitating reconstruction and can be preferred in applicable cases. In the series of a French group of head and neck robotic surgery,^18^ they reported 13 patients with cT2‒T3 tumors out of 23 patients in total (10 were T1), and only 1 patient received reconstruction with free flap. However, they also reported 2 cases of postoperative spondylodiscitis, 1 of which died of a vertebral fracture resulting in spinal cord transection. Therefore, they exclusively recommended free flap reconstruction in patients with extensive resection or history of previous RT to the head and neck region in order to prevent such complications. TORS for hypopharyngeal cancers is relatively new in this field and should be considered in patients with small tumors and good exposure of the hypopharynx. TLM is a treatment modality proven to be safe in laryngeal cancer patients with a good laryngeal exposure and it has been reported to be effective also in posterior pharyngeal wall tumors by Canis^17^ et al. However, TLM for hypopharyngeal tumors presents some difficulties, especially for PPW tumors. It is critical to ensure safe surgical margins distally in the esophagus, which is difficult to acheive with TLM. Another issue to be considered is the assessment of the deep surgical margins.

Hypopharyngeal tumors are often diagnosed in advanced stage, as mentioned above. When surgical treatment is planned for these patients, large resections are being performed considering the risk of submucosal extension and skip lesions. In the presence of PPW tumors, the deep surgical margin is naturally the prevertebral fascia. The patients in our series required extended resection resulting in a large pharyngeal defect, which had to be reconstructed with a free flap. In all of the cases, reconstruction with radial forearm free flap was preferred. It has a thin and pliable structure owing to its fasciocutaneous structure, which makes it ideal for patching the pharyngeal defect, and it also provides flexibility of flap size depending on the defect size. Pedicled flaps likethe pectoralis major myocutaneous flap are too bulky to be placed behind larynx,so they may hinder laryngeal functions. Furthermore, the cosmetic deformity and morbidity at the donor site is less with free tissue transfer. The pedicled flaps may be a secondary choice in case of failure of free flaps. Repair with split thickness grafts is not considered to be a reliable technique for such large defects. Reconstruction with jejunal flaps was recommended by Nakatsuka et al. who stated that less swallowing problems are encountered with this flap.^19^ The necessity of the attendance of a general surgeon to the operation and postoperative abdominal morbidities are the major disadvantages of this technique. The local pedicled flaps which can be harvested from the neck such as supraclavicular or platysmal flap may not be large enough and none of them results in success rates as high as a radial forearm free flap.

Despite the small number of patients, oncological outcomes regarding local control rate was good. It should be noted that most of these patients received combined treatment with adjuvant RT alone (in 4 patients) or adjuvant CRT (in 4 patients). On the other hand, we can comment that 6 of the 10 patients did not receive chemotherapy, which significantly increases toxicity in primary CRT protocols. Choosing surgical treatment primarily provides the advantage of accurate staging of the disease both clinically and pathologically and determining the prognostic factors, which may avoid CT.

The authors prefer to perform routine tracheostomy as a precaution against airway compromise in the postoperative period, as these patients are prone to laryngeal edema. The tracheostomy is retained until the end of the radiotherapy, if the patient is going to receive RT. This has the disadvantage of presence of tracheostomy for a longer time. On the other hand, it is obviously more secure and has the advantage of a shortened hospital stay because the patients are discharged from hospital with tracheostomy, which provides less concern about aspiration. In our study, the decannulation time is longer than the previously published series,^19^, ^20^ but we did not encounter aspiration pneumonia in the early or late postoperative period. The oral intake time was in range of 15–150 days, and all patients regained a regular diet. Large resections for advanced pharyngeal tumors impact laryngeal functions in the early postoperative period, which further deteriorates during adjuvant RT. Additionally, postoperative flap swelling and crusting of the skin surface are thought to be the cause of the swallowing problems after reconstruction with radial forearm free flap.^19^ Nevertheless, the patients regain swallowing functions by the time the edema resolves and this period highly depends on the severity of RT induced toxicity. The prolonged gastrostomy was reported to be significantly associated with T3‒T4 disease at initial diagnosis in a series, but it was found that there was no significant difference between the primary RT/CRT group and the surgical treatment group regarding the gastrostomy or tracheostomy presence beyond 1 year.^15^ In our patients, the swallowing function recovered within a mean time of 75 days and the subjective evaluation of the quality of swallowing by EAT-10 and FOIS was satisfactory.

## Conclusion

Primary surgical treatment of hypopharyngeal tumors located in PPW is still a good therapeutic choice for these patients and does not compromise their swallowing function permanently if pharyngeal reconstruction is performed with a free flap.

## Conflicts of interest

The authors declare no conflicts of interest.
